# Diminished Return of Employment on Ever Smoking Among Hispanic Whites in Los Angeles

**DOI:** 10.1089/heq.2018.0070

**Published:** 2019-04-11

**Authors:** Shervin Assari, Ritesh Mistry

**Affiliations:** ^1^ Department of Psychology, Research and Policy, University of California, Los Angeles (UCLA), Los Angeles, California.; ^2^ BRITE Center for Science, Research and Policy, University of California, Los Angeles (UCLA), Los Angeles, California.; ^3^ Center for Research on Ethnicity, Culture, and Health (CRECH), School of Public Health, University of Michigan, Ann Arbor, Michigan.; ^4^ Department of Psychiatry, University of Michigan, Ann Arbor, Michigan.; ^5^ Department of Health Behavior and Health Education, School of Public Health, University of Michigan, Ann Arbor, Michigan.

**Keywords:** employment, Hispanics, smoking, socioeconomic status (SES)

## Abstract

**Purpose:** According to the Minorities' Diminished Return (MDR) theory, socioeconomic status (SES) indicators such as employment status produce smaller tangible outcomes for racial and ethnic minority groups, however, very limited information exists on such diminished returns for Hispanics. To test whether MDR also holds for the social patterning of smoking behaviors among white adults, this study explored ethnic differences in the association between employment status and ever smoking in a representative sample of adults in Los Angeles.

**Methods:** Los Angeles Family and Neighborhood Survey 2001 included 907 non-Hispanic white and 2117 Hispanic white adults (ages 18 or older). Ethnicity, gender, age, employment status, marital status, immigration status, and ever smoking were measured. Logistic regression models were used for data analysis.

**Results:** In the pooled sample that included both non-Hispanic whites and Hispanic whites, being employed was associated with lower odds of ever smoking, net of covariates. A significant interaction was found between ethnicity and employment status on odds of ever smoking, suggesting a stronger inverse association between employment status and ever smoking for non-Hispanic whites than Hispanic whites. Ethnic specific models showed an inverse association between being employed and ever smoking status for non-Hispanic whites, but not for Hispanic whites.

**Conclusion:** Even among whites, whether or not employment reduces the risk of ever smoking may depend on ethnicity, with Hispanics being at a disadvantage relative to non-Hispanic whites in terms of lower odds of ever smoking from their employment status.

## Introduction

Employment status is one of the most powerful social determinants of health,^1^ as it impacts health behaviors,^2^ mental health,^3^ chronic disease, and life expectancy.^4^ Between various social determinants, employment status may have the strongest effect on mortality risk.^4^ Individuals who lose their jobs show a deterioration in health.^5^ It is, however, unknown whether the association between employment status and health also depends on ethnicity or not.

As the Minorities' Diminished Return (MDR) theory argues,^6,7^ high socioeconomic status (SES) shows a stronger association with health behaviors, mental health, and physical health for whites compared with racial and ethnic minority populations. For example, education attainment has a stronger impact on diet,^8^ sleep,^9^ exercise,^9^ smoking,^10^ alcohol use,^11^ impulse control,^12^ and obesity^13,14^ for whites than blacks. The same pattern is shown for employment status,^15^ as being employed better reduces the risk of mortality for whites relative to blacks.^15^

Although tens of studies have confirmed the relevance of MDR theory to the comparison of non-Hispanic blacks and non-Hispanic whites, very few studies have ever tested the same patterns regarding the effects of Hispanic ethnicity among whites. That means, we know much more about racial differences in health gains due to SES, in comparison to the role that Hispanic ethnicity may play in this regard.

We are only aware of a single study^16^ built on the MDR theory on differential association between family SES and health outcomes between Hispanic whites and non-Hispanic whites. This study used data from the Collaborative Psychiatric Epidemiology Surveys (CPES), 2001–2003, which included 7587 non-Hispanic whites and 3620 Hispanic whites for the associations between education attainment, income, employment, and marital status with self-rated oral health. This study showed that all SES indicators promoted self-rated oral health for non-Hispanic whites; however, none of the same indicators improved the same outcome for Hispanic whites.^16^ Although the results were suggestive that similar patterns may exist for all nonwhite populations, we still need studies that compare Hispanics and non-Hispanics for the health effects of similar SES indicators.

This information is very important because it will determine whether the diminished returns that are seen for blacks^6,7^ can be seen even among whites of different ethnicities; even among whites, being Hispanic may mean a relative disadvantage for gaining health benefits from SES resources.^16^ Such conclusion will suggest that these diminished returns are nonspecific to race and ethnic groups and should be expected for any population that lacks the privilege that non-Hispanic whites enjoy. The policy implication of such conclusion would be the privilege that whites are enjoying is the neglected root cause of health inequity, and the solution to health gaps is that social groups should become equal not only in the access to their resources but also how they are treated by various institutions in the society. Efforts should be made such that the society treats ethnic groups equally and various groups can have identical access to the opportunity structure.

### Aim

The present study examined at the ethnic differences in the association between employment status and ever smoking in a representative sample of adults in Los Angeles. The results are expected to expand the present knowledge on the diminished return of SES for Hispanic whites relative to non-Hispanic whites. If we find such differences, it would mean that even when the skin color is white, ethnicity matters in shaping how SES affects health and health behaviors. This is in continuation of the discussion that for no racial and ethnic minority group, high SES can buy “whiteness,” even when the skin color of the group is white.^17^

## Methods

### Design and setting

The present study had a cross-sectional design. The study used data from wave 1 of the 2000–2001 Los Angeles Family and Neighborhood Survey (L.A.FANS), a representative study of all households in Los Angeles County, CA.^18^

### Ethics

The L.A.FANS study protocol was approved by the RAND Institutional review board (IRB). All participants provided written informed consent.

### Participants and sampling

The L.A.FANS oversampled low-income families/neighborhoods.^18^ The original sample included 2620 individuals in 65 census tracts. We limited our analyses to adults ages 18 years or older, those who had complete data on our variables, and were either non-Hispanic or Hispanic white. The present analysis included 907 non-Hispanic white and 2117 Hispanic white adults.

### Study variables

Variables were selected based on our conceptual model regarding the association between employment status and physical health, so required confounders such as demographic variables (age and gender), immigration status, and other SES indicators (education and marital status) were retained for analyses. All the study constructs were individual-level variables.

#### Main independent variable

This study used employment status as the main independent variable. Participants were asked “Are you currently working?” Current employment status was treated as a dichotomous variable (1 currently working, 0 currently not working).

#### Main dependent variable

Ever smoking was measured by the following item: “Did you ever smoke cigarettes?” Responses were 1 yes or 0 no.

#### Confounders

Our study also included the following sociodemographic variables as confounders. Gender, age, years of education, marital status, and immigration status. Age was operationalized as an interval measure. Gender was treated as a dichotomous measure (1 male 0 female). Education was number of years of schooling. Marital status was operationalized as a dichotomous variable (1 married 0 other status). Immigration status was a dichotomous variable (1 US born, 0 born outside the United States).

### Data analysis

We analyzed the data using Stata version 15.0 (StataCorp LP, College Station, TX) that allowed us to consider sampling weights. Descriptive statistics, bivariate correlations, and logistic regression models were performed. For bivariate correlations, we reported r coefficients from Pearson correlation tests. From our logistic regression models, we reported adjusted regression coefficients (b) and corresponding 95% confidence intervals (CIs), z and *p*-values.

We ran four logistic regression models with sever smoking as the main outcome. *Model 1* and *Model 2* were performed in the pooled sample. *Model 1* did not include the interaction term, while *Model 2* included the ethnicity by employment status interaction as a variable. *Model 3* and *Model 4* were specified in non-Hispanic white and Hispanic white adults, respectively. We tested our main hypothesis in *Model 2* that introduced an interaction term between ethnicity and employment status.

## Results

### Descriptive statistics

The present study included 907 non-Hispanic white and 2117 Hispanic white adults from Los Angeles city. Table 1 describes the overall sample, as well as ethnic groups. As Table 1 shows, different from non-Hispanic whites who were mostly US born, most Hispanic whites were born outside the United States. Non-Hispanic whites were considerably older compared with Hispanic whites (47.68 vs. 37.54; *p*<0.05). Years of education were also significantly higher for non-Hispanic whites in comparison with Hispanic whites (14.97 vs. 10.27; *p*<0.05). Prevalence of ever smoking status was higher for non-Hispanic whites in comparison with Hispanic whites (34.80% vs. 26.77%; *p*<0.05).

**Table 1.** Descriptive Statistics in the Pooled Sample and by Ethnicity

	All (*n*=3024)			Non-Hispanic whites (*n*=907)			Hispanic whites (*n*=2117)		
	%	SE	95% CI	%	SE	95% CI	%	SE	95% CI
Ethnicity									
Non-Hispanic white	49.06	0.02	45.99–52.13	—	—	—	—	—-	—
Hispanic white	50.94	0.02	47.87–54.01	—	—	—	—	—	—
US citizen^*^									
No	30.40	0.01	27.92–33.00	5.07	0.01	3.41–7.47	54.80	0.02	51.15–58.39
Yes	69.60	0.01	67.00–72.08	**94.93**	0.01	92.53–96.59	45.20	0.02	41.61–48.85
Gender									
Female	50.59	0.02	47.53–53.64	50.66	0.03	45.69–55.62	50.52	0.02	46.90–54.12
Male	49.41	0.02	46.36–52.47	49.34	0.03	44.38–54.31	49.48	0.02	45.88–53.10
Marital status^*^									
Others	49.13	0.02	46.13–52.13	44.12	0.03	39.27–49.09	53.86	0.02	50.35–57.32
Married	50.87	0.02	47.87–53.87	**55.88**	0.03	50.91–60.73	46.14	0.02	42.68–49.65
Employed									
No	33.65	0.01	30.81–36.61	35.37	0.02	30.73–40.32	31.98	0.02	28.79–35.36
Yes	66.35	0.01	63.39–69.19	64.63	0.02	59.68–69.27	68.02	0.02	64.64–71.21
Ever smoking^*^									
No	69.38	0.02	66.15–72.44	65.20	0.03	59.70–70.33	73.23	0.02	69.61–76.56
Yes	30.62	0.02	27.56–33.85	**34.80**	0.03	29.67–40.30	26.77	0.02	23.44–30.39
	**Mean**	**SE**	**95% CI**	**Mean**	**SE**	**95% CI**	**Mean**	**SE**	**95% CI**
Age^*^	42.58	0.60	41.40–43.76	**47.68**	0.98	45.76–49.60	37.54	0.61	36.34–38.74
Education (years)^*^	12.61	0.13	12.35–12.87	**14.97**	0.14	14.69–15.26	10.27	0.17	9.94–10.60

^*^ *p*<0.05 for comparison of non-Hispanic and Hispanic whites. Bold number is significantly larger than the other group. Source: wave 1 of the 2000–2001 Los Angeles Family and Neighborhood Survey (L.A.FANS).

### Logistic regression in the overall sample

Table 2 summarizes the results of two logistic regressions in the pooled sample, with ever smoking as the main outcome. *Model 1*, which did not include any interaction term, suggested that being employed is associated with lower odds of ever smoking in the pooled sample (OR=0.65, 95% CI=0.45–0.92), net of covariates. *Model 2* showed an interaction between ethnicity and employment status (b=2.65, 95% CI=1.41–4.97), suggesting stronger protective effects of being employed against odds of ever smoking status for non-Hispanic whites than Hispanic whites.

**Table 2.** Summary of Logistic Regression Models in the Pooled Sample

	Model 1 in all (*n*=3024)					Model 2 in all (*n*=3024)				
	OR	SE	95% CI	Z	*p*	OR	SE	95% CI	Z	*p*
Ethnicity (Hispanic)	**0.66**	**0.14**	**0.44–0.98**	**−2.05**	**0.041**	**0.34**	**0.10**	**0.19–0.62**	**−3.59**	**0.000**
US citizen	1.28	0.25	0.87–1.89	1.24	0.214	1.34	0.27	0.91–1.97	1.46	0.145
Age	1.00	0.01	0.99–1.01	0.38	0.703	1.00	0.01	0.99–1.01	−0.04	0.965
Married	0.82	0.13	0.61–1.11	−1.26	0.208	0.85	0.13	0.63–1.15	−1.03	0.304
Male	**2.04**	**0.33**	**1.49–2.81**	**4.42**	**0.000**	**1.99**	**0.32**	**1.45–2.73**	**4.26**	**0.000**
Education (years)	**0.96**	**0.02**	**0.92–1.00**	**−1.90**	**0.057**	**0.95**	**0.02**	**0.91–0.99**	**−2.22**	**0.026**
Employed	**0.65**	**0.12**	**0.45–0.92**	**−2.40**	**0.017**	**0.41**	**0.11**	**0.24–0.69**	**−3.38**	**0.001**
Education (years)×Ethnicity (Hispanic)						**2.65**	**0.85**	**1.41–4.97**	**3.02**	**0.003**
Intercept	0.73	0.33	0.30–1.79	−0.69	0.493	1.14	0.55	0.44–2.95	0.28	0.780

Bold numbers are statistically significant. Source: wave 1 of the 2000–2001 Los Angeles Family and Neighborhood Survey (L.A.FANS).

OR, adjusted odds ratio; SE, standard error.

### Logistic regression by ethnicity

As Table 3 also shows, in ethnic-stratified models, being employed was linked to lower odds of ever smoking for non-Hispanic whites (b=0.42, 95% CI=0.23–0.75) (*Model 3*), but not for Hispanic whites (b=1.06, 95% CI=0.68–1.64) (*Model 4*).

**Table 3.** Summary of Logistic Regression Models by Ethnicity

	Model 3 in non-Hispanic whites (*n*=907)					Model 4 in Hispanic whites (*n*=2117)				
	OR	SE	95% CI	Z	*p*	OR	SE	95% CI	Z	*p*
US citizen	2.69	2.09	0.59–12.36	1.28	0.202	1.02	0.21	0.67–1.53	0.07	0.943
Age	1.00	0.01	0.99–1.02	0.25	0.801	1.00	0.01	0.99–1.02	0.24	0.812
Married	0.87	0.22	0.53–1.43	−0.56	0.576	0.85	0.16	0.59–1.22	−0.88	0.378
Male	**2.46**	**0.62**	**1.50–4.01**	**3.58**	**0.000**	**1.67**	**0.34**	**1.12–2.50**	**2.50**	**0.013**
Education (years)	**0.89**	**0.04**	**0.81–0.96**	**−2.83**	**0.005**	0.99	0.02	0.95–1.04	−0.29	0.774
Employed	**0.42**	**0.13**	**0.23–0.75**	**−2.91**	**0.004**	1.06	0.24	0.68–1.64	0.26	0.797
Intercept	1.38	1.26	0.23–8.28	0.36	0.722	**0.30**	**0.13**	**0.13–0.70**	**−2.78**	**0.006**

Bold numbers are statistically significant. Source: wave 1 of the 2000–2001 Los Angeles Family and Neighborhood Survey (L.A.FANS).

## Discussion

In a representative sample of Los Angeles white adults, employment status was differently associated with ever smoking status across ethnic groups, with Hispanics being at a relative disadvantage compared with non-Hispanics in gaining such protective effects from their employment status on lifetime smoking.

The findings reported here extend the MDR theory^6,7^ from a literature almost exclusively focused on race (comparison of blacks and whites) to ethnicity (comparison of Hispanics and non-Hispanics). As mentioned before, research has shown that non-Hispanic blacks gain less from their SES indicators compared with nonwhites. The result is in orchestra with the one study that built on MDR on Hispanic ethnicity, on SES and self-rated oral health.^16^ Effect of employment^15^ on mortality is stronger for non-Hispanic whites than non-Hispanic blacks. Education attainment^19^ also better reduces mortality risk for non-Hispanic whites than non-Hispanic blacks. High income better reduces the number of chronic diseases for non-Hispanic whites than non-Hispanic blacks.^20^ For example, income reduces the risk of childhood asthma for non-Hispanic whites than non-Hispanic blacks.^21^

These differential effects are not specific to smoking behaviors as they are also shown for mental health outcomes such as depression^22–24^ and suicide.^25^ Similarly, both education^26^ and income^22–24^ better reduce risk of depression for non-Hispanic blacks relative to non-Hispanic whites. Across age groups, including children,^13,27^ youth,^28–30^ adults,^10^ or older adults,^9^ high SES seems to be associated with better health status for whites than nonwhites.

This study suggests that it is not the nonwhite skin color, but the lack of privileges of the non-Hispanic whites that reduces health gain from SES for nonwhite groups. Even ethnicity reduces the gain that follows SES among ethnically diverse sample of whites. These diminished returns (including the diminished return of employment status) among minorities in the United States can be attributed to the pervasive racism and discrimination in the US society.^6,23,24,31,32^ SES does not similarly prevent stress for whites and nonwhites.^6,23,24,31,33^ For nonwhites, an increase in SES means an increase in contact with whites,^23,32^ which is associated with an increase in interpersonal discrimination.^30,31^ High SES adolescent minorities more frequently attend majority white schools.^23^ High SES adult minorities work in predominantly white work places.^32^ Discrimination has several negative health effects^34^ and also reduces the health gain of SES.^35^

Preferences and practices of the labor market also play a major role in reducing the health gains of SES for minorities, particularly employment status.^36^ Due to the labor market preferences and practices, the very same employment status means more income for whites than other ethnic groups,^37^ a pattern that may explain why employment is associated with a larger increase in the life expectancy for non-Hispanic whites than nonwhites.^15^ High education better helps whites than nonwhites to escape poverty.^38^

In a 10-year cohort study, higher educational attainment at baseline was associated with a larger gain in income for non-Hispanic whites but not for non-Hispanic blacks.^37^ At the same time, neither education nor income was associated with an increase in positive affect for non-Hispanic blacks; however, both of these SES indicators resulted in a positive affect for whites.^37^ In another study with a nationally representative sample, income levels mediated the diminished return of education on self-rated health for non-Hispanic blacks in comparison with non-Hispanic whites (in press). MDRs are stronger for non-Hispanic blacks.

Lifetime smoking is high in employed Hispanic whites. Depending on the type of occupation, employment differently reduces exposure to stress and environmental risk factors for various social groups. At the same time, upward social mobility is associated with extra psychosocial costs for nonwhites than whites.^35^ Even for the families and individuals who successfully climb the social ladder, high social status is not similarly rewarding in terms of life conditions, earned income, purchasing power, access to social power, and health status, neither for the individual nor for the family of the person.^12,14,29,38^

Weaker effects of SES on health behaviors such as smoking at least, in part, explain why SES indicators better reduce risk of chronic disease and mortality for whites than nonwhites. The presence of SES resources has a larger impact on lowering high-risk behaviors such as poor diet,^8^ poor sleep,^9^ low exercise,^9^ smoking,^10^ alcohol use,^11^ impulsivity,^12^ and obesity^13,14^ for whites than nonwhites. These unhealthy behaviors increase risk of metabolic disorders such as chronic respiratory disease, heart disease, obesity, hypertension, diabetes, and stroke.^39^

Subsequently, poor behavior [meaning, as a consequence of the prior effects just mentioned] may be a mechanism explaining why SES indicators such as employment status better reduce risk of chronic medical disease^21^ for whites than racial and ethnic minority groups. Future research should test whether diminished return of SES on health behaviors explains diminished return of SES on depression and chronic disease for racial and ethnic minority populations.

### Implications

The results suggest that while employed non-Hispanic whites are at a lower risk of lifetime smoking, employed Hispanic whites are still at high risk of ever smoking. That suggests a high need for smoking cessation programs and interventions in occupations that are predominantly Hispanic whites. Such investment may not, however, be as needed for occupations that are non-Hispanic whites, given the protection non-Hispanic whites gain from employment. This is informative for policy makers and program planners and suggests high SES does not similarly signal health status across ethnic groups.

In other words, although overall, employed individuals need less investment for prevention and education of smoking, this is particularly true for non-Hispanic whites and less for Hispanic whites. Employment status does not convey much information regarding lifetime smoking status for Hispanic whites. This is another reason that policy and public health programs benefit from a tailored approach that considers the specific needs of each subsection of the society.^40^

### Limitations

The present study had some methodological limitations. First, due to a cross-sectional design, any causal inferences should be avoided. While unemployment may operate as a risk factor for poor health, poor health may also cause unemployment, downward social mobility, and low SES. Second, only a handful of confounders were included in this study. This is a threat to the validity of the findings as the employment and health association may have more confounders compared with other SES indicators. Third, all the study variables here were individual level. Several contextual factors such as area SES as well as density of job opportunities in the neighborhood may confound the SES and health link by ethnicity.^41^ Future research should study contextual and environmental factors that operate as underlying mechanisms for diminished returns of SES for Hispanics and other minorities. There is also a need to study the role of income, financial difficulty, food insecurity, and insurance status on these effects.^42^

Fourth, the sample size was imbalanced between the ethnic groups. Differential sample size may result in different statistical power, especially for ethnic-stratified models. We are, however, not very concerned about the differential statistical power because the association of interest was missing in the group with a larger sample size. Fifth, our ethnic groups were not comparable in age, which shapes the pattern of lifetime smoking.

Sixth, the outcome of the present study was measured using a single item, which is subject to recall bias. Self-report measures of smoking may be prone to different levels of measurement error across ethnic groups. In addition, our outcome was lifetime rather than current smoking status. Last but not least, we did not consider the within-Hispanics heterogeneity. Hispanic groups differ in SES, culture, historical background, behaviors, and health status. Studies should replicate these findings in more homogeneous groups as well as subgroups based on nativity and immigration status. Future research should also attempt to replicate the results reported here using longitudinal data, with employment and other SES indicators measured at multiple observations. More research is also needed on current smoking as well as the dosage of smoking.

## Conclusion

In summary, although being employed is associated with lower odds of lifetime smoking overall, this protection can be detected for non-Hispanic whites, but not for Hispanic whites. This finding supports the application of MDR theory for epidemiological studies that wish to understand the social patterning of health risk behaviors in ethnically diverse populations.

## Abbreviations Used

MDR: Minorities' Diminished Return
SES: socioeconomic status

## References

[1] Wellman RJ, Contreras GA, Dugas EN, et al. Determinants of sustained binge drinking in young adults. Alcohol Clin Exp Res. 2014;38:1409–1415 24512139 10.1111/acer.12365

[2] Heikkila K, Fransson EI, Nyberg ST, et al. Job strain and health-related lifestyle: findings from an individual-participant meta-analysis of 118,000 working adults. Am J Public Health. 2013;103:2090–2097 23678931 10.2105/AJPH.2012.301090 PMC4984954

[3] Mandal B, Ayyagari P, Gallo WT Job loss and depression: the role of subjective expectations. Soc Sci Med. 2011;72:576–583 21183267 10.1016/j.socscimed.2010.11.014 PMC3684950

[4] Rogot E, Sorlie PD, Johnson NJ Life expectancy by employment status, income, and education in the National Longitudinal Mortality Study. Public Health Rep. 1992;107:457–461 1641443 PMC1403677

[5] Noelke C, Beckfield J Recessions, job loss, and mortality among older US adults. Am J Public Health. 2014;104:e126–e134 25211731 10.2105/AJPH.2014.302210 PMC4202979

[6] Assari S Health Disparities due to Diminished Return among Black Americans: Public Policy Solutions. Soc Iss Policy Rev. 2018;12:112–145

[7] Assari S Unequal Gain of Equal Resources across Racial Groups. Int J Health Policy Manag. 2017;7:1–9 29325397 10.15171/ijhpm.2017.90 PMC5745862

[8] Assari S, Lankarani M Educational Attainment Promotes Fruit and Vegetable Intake for Whites but Not Blacks. J. 2018;1:29–41 10.3390/j1010005 PMC6914217 31844842

[9] Assari S, Nikahd A, Malekahmadi MR, et al. Race by Gender Group Differences in the Protective Effects of Socioeconomic Factors Against Sustained Health Problems Across Five Domains. J Racial Ethn Health Disparities. 2017;4:884–894 10.1007/s40615-016-0291-3 27753050

[10] Assari S, Mistry R Educational Attainment and Smoking Status in a National Sample of American Adults; Evidence for the Blacks' Diminished Return. Int J Environ Res Public Health. 2018;15:pii: 10.3390/ijerph15040763 PMC5923805 29659482

[11] Assari S, Lankarani MM Education and Alcohol Consumption among Older Americans; Black-White Differences. Front Public Health. 2016;4:67 27148514 10.3389/fpubh.2016.00067 PMC4838609

[12] Assari S, Caldwell CH, Mincy R Family Socioeconomic Status at Birth and Youth Impulsivity at Age 15; Blacks' Diminished Return. Children (Basel). 2018;5:pii: 10.3390/children5050058 PMC5977040 29724004

[13] Assari S Family Income Reduces Risk of Obesity for White but Not Black Children. Children (Basel). 2018;5:pii: 10.3390/children5060073 PMC6025246 29890778

[14] Assari S, Thomas A, Caldwell CH, et al. Blacks' Diminished Health Return of Family Structure and Socioeconomic Status; 15 Years of Follow-up of a National Urban Sample of Youth. J Urban Health. 2018;95:21–35 29230628 10.1007/s11524-017-0217-3 PMC5862702

[15] Assari S Life Expectancy Gain Due to Employment Status Depends on Race, Gender, Education, and Their Intersections. J Racial Ethn Health Disparities. 2018;5:375–386 28634876 10.1007/s40615-017-0381-x PMC6392452

[16] Assari S Socioeconomic Status and Self-Rated Oral Health; Diminished Return among Hispanic Whites. Dent J (Basel). 2018;6:pii: 10.3390/dj6020011 PMC6023433 29695074

[17] Pearson JA CAN'T BUY ME WHITENESS: New Lessons from the Titanic on Race, Ethnicity, and Health. Du Bois Rev 2008;5:27–47

[18] Anne RP, and Narayan S Los Angeles Family and Neighborhood Study (L.A.FANS). Ann Arbor, MI: Inter-university Consortium for Political and Social Research [distributor], 2013-09-18. Available at 10.3886/ICPSR22940.v4 Accessed 121, 2018

[19] Assari S, Lankarani MM Does Multi-morbidity Mediate the Effect of Socioeconomics on Self-rated Health? Cross-country Differences. Int J Prev Med. 2015;6:85 26445632 10.4103/2008-7802.164413 PMC4587073

[20] Assari S Number of Chronic Medical Conditions Fully Mediates the Effects of Race on Mortality; 25-Year Follow-Up of a Nationally Representative Sample of Americans. J Racial Ethn Health Disparities. 2017;4:623–631 27440120 10.1007/s40615-016-0266-4 PMC6662183

[21] Martin LM, Leff M, Calonge N, et al. Validation of self-reported chronic conditions and health services in a managed care population. Am J Prev Med. 2000;18:215–218 10722987 10.1016/s0749-3797(99)00158-0

[22] Assari S, Lankarani MM Race and Urbanity Alter the Protective Effect of Education but not Income on Mortality. Front Public Health. 2016;4:100 27242992 10.3389/fpubh.2016.00100 PMC4873510

[23] Assari S The Benefits of Higher Income in Protecting against Chronic Medical Conditions Are Smaller for African Americans than Whites. Healthcare (Basel). 2018;6: pii: 10.3390/healthcare6010002 PMC5872209 29315227

[24] Assari S, Moghani Lankarani M Poverty Status and Childhood Asthma in White and Black Families: National Survey of Children's Health. Healthcare (Basel). 2018;6: pii: 10.3390/healthcare6020062 PMC6023379 29895767

[25] Assari S, Caldwell CH High Risk of Depression in High-Income African American Boys. J Racial Ethn Health Disparities. 2018;5:808–819 28842841 10.1007/s40615-017-0426-1 PMC6556394

[26] Assari S Does School Racial Composition Explain Why High Income Black Youth Perceive More Discrimination? A Gender Analysis. Brain Sci. 2018;8:140 10.3390/brainsci8080140 PMC6119879 30061476

[27] Assari S, Lankarani MM, Caldwell CH Does Discrimination Explain High Risk of Depression among High-Income African American Men? Behav Sci (Basel). 2018;8: pii: 10.3390/bs8040040 PMC5946099 29671796

[28] Assari S Ethnic and Gender Differences in Additive Effects of Socio-economics, Psychiatric Disorders, and Subjective Religiosity on Suicidal Ideation among Blacks. Int J Prev Med. 2015;6:53 26180624 10.4103/2008-7802.158913 PMC4498310

[29] Assari S Combined Racial and Gender Differences in the Long-Term Predictive Role of Education on Depressive Symptoms and Chronic Medical Conditions. J Racial Ethn Health Disparities. 2017;4:385–396 27270925 10.1007/s40615-016-0239-7

[30] Assari S, Hani N Household Income and Children's Unmet Dental Care Need; Blacks' Diminished Return. Dent J (Basel). 2018;6:pii: 10.3390/dj6020017 PMC6023279 29867015

[31] Assari S, Caldwell CH Low Family Support and Risk of Obesity among Black Youth: Role of Gender and Ethnicity. Children (Basel). 2017;4:pii: 10.3390/children4050036 PMC5447994 28498351

[32] Assari S, Caldwell CH, Mincy RB Maternal Educational Attainment at Birth Promotes Future Self-Rated Health of White but Not Black Youth: A 15-Year Cohort of a National Sample. J Clin Med. 2018;7:pii: 10.3390/jcm7050093 PMC5977132 29723957

[33] Assari S, Gibbons FX, Simons R Depression among Black Youth; Interaction of Class and Place. Brain Sci. 2018;8:pii: 10.3390/brainsci8060108 PMC6025590 29895752

[34] Assari S, Gibbons FX, Simons RL Perceived Discrimination among Black Youth: An 18-Year Longitudinal Study. Behav Sci (Basel). 2018;8:pii: 10.3390/bs8050044 PMC5981238 29702587

[35] Assari S, Moghani Lankarani M Workplace Racial Composition Explains High Perceived Discrimination of High Socioeconomic Status African American Men. Brain Sci. 2018;8:pii: 10.3390/brainsci8080139 PMC6120025 30060492

[36] Assari S, Caldwell CH Social Determinants of Perceived Discrimination among Black Youth: Intersection of Ethnicity and Gender. Children (Basel). 2018;5:pii: 10.3390/children5020024 PMC5835993 29462893

[37] Mays VM, Cochran SD, Barnes NW Race, race-based discrimination, and health outcomes among African Americans. Annu Rev Psychol. 2007;58:201–225 16953796 10.1146/annurev.psych.57.102904.190212 PMC4181672

[38] Hudson DL, Bullard KM, Neighbors HW, et al. Are benefits conferred with greater socioeconomic position undermined by racial discrimination among African American men? J Mens Health. 2012;9:127–136 22707995 10.1016/j.jomh.2012.03.006 PMC3371660

[39] Browne I, Misra J The intersection of gender and race in the labor market. Annu Rev Sociol. 2003;29:487–513

[40] Assari S, Preiser B, Kelly M Education and Income Predict Future Emotional Well-Being of Whites but Not Blacks: A Ten-Year Cohort. Brain Sci. 2018;8:pii: 10.3390/brainsci8070122 PMC6070982 29966278

[41] Assari S Parental Education Better Helps White than Black Families Escape Poverty: National Survey of Children's Health. Economies. 2018;6:30

[42] Lindtner C, Scherer T, Zielinski E, et al. Binge drinking induces whole-body insulin resistance by impairing hypothalamic insulin action. Sci Transl Med. 2013;5:170ra14 10.1126/scitranslmed.3005123 PMC3740748 23363978

